# Clinical Memoranda from the Surgical Clinic at the Sisters’ of Charity Hospital

**Published:** 1894-02

**Authors:** Herman Mynter

**Affiliations:** Professor of Surgery Niagara University and Surgeon to the Sisters’ Hospital


### CLINICAL MEMORANDA FROM THE SURGICAL CLINIC AT THE SISTERS' OF CHARITY HOSPITAL.

By HERMAN MYNTER, M. D.

Professor of Surgery Niagara University and Surgeon to the Sisters' Hospital.

### SYNOPSIS OF ONE YEAR'S WORK IN THE HOSPITAL.

During the year 1893, 300 surgical patients, exclusive of gynecological and ophthalmological cases, were admitted to the Sisters' of Charity Hospital. Of these, 265 cases were treated in my service, the rest in the services of Drs. Mickle and Heath. While this number is somewhat less than in former years, particularly on account of the largely increased number of injured persons from the manufacturing districts who seek admittance to the downtown emergency hospitals, the work has in reality very much increased, and a much better class of patients, both from the town and surrounding country, have availed themselves of the, at least in Western New York, unsurpassed facilities of the Sisters' Hospital. The old repugnance of former years against hospitals begins to die out, and the community has learned at last that nowhere can a serious operation be performed with such ease to the surgeon and safety and comfort to the patients as in a well-conducted hospital. That such is the case is best seen by the large number of private patients constantly found in the hospital, or waiting for vacant rooms. The hospital has been crowded during the whole year, 1,755 patients having been treated, *vs.* 1,678 in 1892, and in the coming year we hope the other half of the hospital will be built. It is the intention of the Sisters to finish this largely for private patients alone, confining the wards, medical and surgical, to the old building. During the year, the new aseptic clinic and operating room, built in marble and cement, has been finished at large expense. It is unsurpassed in the State of New York, outside New York City; thoroughly aseptic, and supplied with every surgical convenience and appliance. Every wound treated there has healed by first intention.

By looking over the list of operations performed, 183 in number, it will be seen how many have been of the class called major operations, and how small the mortality has been, particularly of those on whom large operations have been performed.

Two cases died of appendicitis, with general peritonitis, having been sick, respectively, eight and six days before being sent to the hospital. In both cases, perforation of the appendix, from gangrene, with septic peritonitis, was found. Two similar cases, operated on second and third day, recovered promptly by laparatomy.

Two cases died after laparatomy, sent to the hospital for obstruction of the bowels. In one case, the lesion was found to be peritonitis from ulcerative gangrene of the cecum; in the other, extrauterine pregnancy, with rupture of the sac, and enormous intra-abdominal hematocele.

Three cases died of fracture of theb ase of skull, with contusion and laceration of brain. One case of double amputation of the legs died shortly after admission. He had laid during the whole night, with his legs crushed by a railroad, in a ditch near Tonawanda.

One case died of pyemia, from a deep-seated abscess on the neck; another from similar cause, from acute infectious osteomyetitis.

One old man, with an enormous inguinal hernia, reaching almost to the knee, died on the third day after Bassini's operation, from fatty heart. There was no symptom of peritonitis present.

A particularly deplorable case was that of a young man, who entered the hospital on account of acute hydronephrosis. A simple exploratory incision was made in the lumbar region, and a floating kidney found, which, by twisting, had closed the ureter. The kidney was fastened by the usual method and the wound plugged with iodoform-gauze. Much to our surprise and regret, he died of shock on the second day. No *post-mortem* was allowed. This is the second case only in which death has occurred as the direct result of an operation during my connection with the Sisters' Hospital.

| DISEASES OF BONES AND JOINTS. | No. Treated. | Recovered. | Improved. | Not Improved. | Died. | Convalescent in Hospital. |
|---|---|---|---|---|---|---|
| Abscess of bone | 1 | 1 | . . | . . | . . | . . |
| Tuberculous arthritis | 6 | 5 | . . | . . | 1 | . . |
| Caries | 6 | 2 | 2 | . . | . . | 2 |
| Crushes | 7 | 7 | . . | . . | . . | . . |
| Coxitis | 8 | 1 | 4 | . . | . . | 3 |
| Fractures, simple | 34 | 27 | 2 | . . | . . | 5 |
| Fractures, compound | 3 | 2 | . . | . . | 1 | . . |
| Fractures, comminuted | 3 | 2 | . . | . . | 1 | . . |
| Fractures, of pelvis | 1 | 1 | . . | . . | . . | . . |
| Fractures, of patella | 2 | 2 | . . | . . | . . | . . |
| Fractures, of skull | 3 | 3 | . . | . . | . . | . . |
| Fractures, of base of skull | 4 | 1 | . . | . . | 3 | . . |
| Necrosis | 5 | 4 | . . | . . | . . | 1 |
| Sprains | 3 | 3 | . . | . . | . . | . . |
| Synovitis, chronic | 2 | . . | 1 | . . | . . | 1 |
| Synovitis, tuberculous | 3 | 1 | 2 | . . | . . | . . |
| Anchylosis | 4 | 4 | . . | . . | . . | . . |
| Pes valgus dolorosus | 1 | 1 | . . | . . | . . | . . |
| Dislocation, shoulder | 2 | 2 | . . | . . | . . | . . |
| Dislocation, thumb | 1 | 1 | . . | . . | . . | . . |
| Dislocation, elbow | 1 | 1 | . . | . . | . . | . . |
| Periostitis, simple | 7 | 6 | . . | . . | . . | 1 |
| Epiphysitis, tuberculous | 5 | 5 | . . | . . | . . | . . |
| Osteomyelitis, acute infectious | 3 | 2 | . . | . . | 1 | . . |
| Osteomyelitis, chronic | 5 | 5 | . . | . . | . . | . . |
| Bowlegs | 1 | 1 | . . | . . | . . | . . |
| Vicious union of fracture of femur and tibia | 2 | 2 | . . | . . | . . | . . |
| AFFECTIONS OF SOFT PARTS. | | | | | | |
| Abscesses, acute | 10 | 9 | . . | . . | 1 | . . |
| Psoas abscesses | 4 | 4 | . . | . . | . . | . . |
| Diffuse cellulitis | 5 | 5 | . . | . . | . . | . . |
| Burns and scalds | 4 | 2 | 1 | . . | . . | 1 |
| Syphilis, primary | 3 | . . | 2 | . . | . . | 1 |
| Syphilis, secondary | 4 | . . | 4 | . . | . . | . . |
| Syphilis, tertiary | 4 | . . | 3 | . . | 1 | . . |
| Ulcers, varicose | 14 | 13 | 1 | . . | . . | . . |
| Wounds, lacerated | 5 | 5 | . . | . . | . . | . . |
| Wounds, incised | 2 | 2 | . . | . . | . . | . . |
| Hernia, Inguinal | 8 | 4 | . . | 1 | 1 | 2 |

| DISEASES OF BONES AND JOINTS. | No. Treated. | Recovered. | Improved. | Not Improved. | Died. | Convalescent in Hospital. |
|---|---|---|---|---|---|---|
| Hernia, femoral | 3 | 3 | | | | |
| Urinary calculus | 1 | 1 | | | | |
| Foreign body (catheter) in bladder | 1 | 1 | | | | |
| Acute myositis | 1 | 1 | | | | |
| Gonorrhea | 1 | | 1 | | | |
| Hydrocele | 1 | 1 | | | | |
| Hematocele | 1 | 1 | | | | |
| Varicocele | 1 | 1 | | | | |
| Epididymitis, gonorrhoica | 2 | 2 | | | | |
| Epididymitis, tuberculosa | 1 | 1 | | | | |
| Strictura, urethra | 6 | 3 | 3 | | | |
| Strictura, recti | 1 | | 1 | | | |
| Pyonephrosis, from tuberculous kidney | 1 | | | | 1 | |
| Hydronephrosis | 2 | 1 | | | 1 | |
| Urinary fistula | 2 | 2 | | | | |
| Floating kidney | 1 | 1 | | | | |
| Obstruction, bowels | 2 | 1 | | | 1 | |
| Appendicitis | 8 | 5 | 1 | | 2 | |
| Gastritis | 2 | 2 | | | | |
| Cleft palate | 2 | 2 | | | | |
| Contusions | 18 | 18 | | | | |
| Congelation | 1 | 1 | | | | |
| Mastitis | 1 | 1 | | | | |
| Epilepsy | 3 | | 3 | | | |
| Ulcerating amputation stump | 2 | 2 | | | | |
| Senile gangrene | 1 | | 1 | | | |
| Aneurism, ext. carotid | 1 | 1 | | | | |
| TUMORS. | | | | | | |
| Scirrhus | 7 | 7 | | | | |
| Sarcoma | 7 | 2 | 3 | | 1 | 1 |
| Epithelioma | 2 | 1 | | | 1 | |
| Neuroma | 1 | 1 | | | | |
| Lipoma | 2 | 2 | | | | |
| Villous cancer of the bladder | 1 | | | | 1 | |
| Fibroma | 2 | 2 | | | | |
| Hematoma | 2 | 2 | | | | |
| Lymphadenoma | 2 | 2 | | | | |
| Ganglion bursa semimembranosa | 1 | 1 | | | | |
| Hygroma of neck | 2 | 2 | | | | |
| Atheroma | 1 | 1 | | | | |
| Gunshot wound of chest | 1 | 1 | | | | |
| Lymphangitis | 1 | | | | 1 | |
| Clavus | 1 | 1 | | | | |
| Abscess of brain | 1 | 1 | | | | |
| Extrauterine pregnancy | 1 | | | | 1 | |
| Carbunculus dorsi | 1 | 1 | | | | |
| Subdural hemorrhage | 1 | 1 | | | | |
| Pleurisy | 1 | | | | | 1 |
| Empyema | 1 | 1 | | | | |
| Harelip | 1 | 1 | | | | |
| Ectropion | 1 | 1 | | | | |
| Tuberculous glands | 2 | 2 | | | | |
| Hemorrhoids | 1 | 1 | | | | |
| Fistula in ano | 3 | 3 | | | | |
| Total | 300 | 225 | 35 | 1 | 20 | 19 |

| OPERATIONS PERFORMED. | No. Treated. | Cured. | Improved. | Not Improved. | Died. | Convalescent in Hospital. |
|---|---|---|---|---|---|---|
| DEFORMITIES— | | | | | | |
| Plastic operations | 3 | 3 | | | | |
| Tenotomy | 1 | 1 | | | | |
| Osteotomy, linear | 2 | 2 | | | | |
| Osteoclasis | 2 | 2 | | | | |
| Brissement forcé for anchylosis | 4 | 3 | 1 | | | |
| Harelip operation | 1 | 1 | | | | |
| Staphyloraphy | 2 | 2 | | | | |
| AMPUTATIONS. | | | | | | |
| Arm | 4 | 4 | | | | |
| Knee | 1 | 1 | | | | |
| Leg (Sedillot) | 7 | 5 | | | 1 | 1 |
| Leg (Symes) | 1 | 1 | | | | |
| RESECTIONS. | | | | | | |
| Knee | 2 | 1 | | | 1 | |
| Elbow | 3 | 3 | | | | |
| Hip | 1 | | 1 | | | |
| Shoulder | 1 | 1 | | | | |
| Maxilla sup. | 1 | 1 | | | | |
| Of rib, for empyema | 1 | 1 | | | | |
| Arthrotomy of elbow, for anchylosis | 1 | 1 | | | | |
| TREPHINING. | | | | | | |
| For fracture skull | 6 | 3 | | | 3 | |
| For epilepsy | 3 | | 3 | | | |
| For intradural hemorrhage | 1 | 1 | | | | |
| For abscess of brain | 1 | 1 | | | | |
| For acute infectious osteomyelitis | 2 | 1 | | | 1 | |
| For chronic osteomyelitis | 5 | 5 | | | | |
| Sequestrotomy | 10 | 10 | | | | |
| Scraping carious bone | 4 | 3 | | | | 1 |
| Removal tuberculous osteitic focus | 4 | 4 | | | | |
| TUMORS REMOVED. | | | | | | |
| Scirrhus of breast | 6 | 6 | | | | |
| Sarcoma of breast | 2 | 1 | | | 1 | |
| Sarcoma of neck | 2 | 1 | 1 | | | |
| Fibroma | 2 | 2 | | | | |
| Neuroma | 1 | 1 | | | | |
| Lipoma | 2 | 2 | | | | |
| Vaginal hysterectomy, for cancer uteri | 1 | 1 | | | | |
| Inflamed lymphatic glands | 4 | 4 | | | | |
| Hygroma of neck | 2 | 2 | | | | |
| Ganglion bursa semimembranosa | 1 | 1 | | | | |
| Tuberculous epididymitis | 1 | 1 | | | | |
| OTHER OPERATIONS. | | | | | | |
| Appendicitis, laparatomy | 4 | 2 | | | 2 | |
| Appendicitis, extraperitoneal operation | 1 | 1 | | | | |
| Laparatomy, for obstruction | 1 | | | | 1 | |
| Laparatomy, extrauterine pregnancy | 1 | | | | 1 | |
| Herniotomy, for strangulation | 3 | 3 | | | | |
| Herniotomy, radical cure (Bassini) | 7 | 5 | | | 1 | 1 |
| Cystotomy, suprapubic | 1 | | 1 | | | |
| Cystotomy, perineal | 2 | 1 | 1 | | | |
| Litholapaxy | 1 | 1 | | | | |
| Urethrotomy, internal | 3 | 3 | | | | |

| OPERATIONS PERFORMED. | No. Treated. | Cured. | Improved. | Not Improved. | Died. | Convalescent in Hospital. |
|---|---|---|---|---|---|---|
| Urethrotomy, external | 1 | 1 | | | | |
| Stricture, dilated | 3 | | 3 | | | |
| Castration | 1 | 1 | | | | |
| Nephrectomy | 1 | | 1 | | | |
| Nephrotomy | 2 | 2 | | | | |
| Floating kidney, sutured | 2 | 1 | | | 1 | |
| Varicocele | 1 | 1 | | | | |
| Hematocele, decortication | 1 | 1 | | | | |
| Hydrocele | 1 | 1 | | | | |
| Fistula ani | 3 | 3 | | | | |
| Hemorrhoids | 1 | 1 | | | | |
| Wiring fractured patella | 2 | 2 | | | | |
| Abscess, opened | 18 | 15 | | | 1 | 2 |
| Cold abscess | 1 | 1 | | | | |
| Psoas abscess | 4 | 4 | | | | |
| Aspiration, pleuritic | 1 | | | | | 1 |
| Aspiration, knee-joint | 2 | | 2 | | | |
| Empyema, operation | 1 | 1 | | | | |
| Skin-grafting | 6 | 4 | | | | 2 |
| Secondary suture | 3 | 3 | | | | |
| Iodoform injection in tuberculous joints | 10 | 4 | 3 | | | 3 |
| Injection toxic products of erysipelas, for sarcoma | 1 | | 1 | | | |
| Extirpation carbuncle | 1 | 1 | | | | |
| Total | 189 | 146 | 18 | | 14 | 11 |

